# The Utility of Total Thrombus‐Formation Analysis System (T‐TAS) in the Thrombosis and Hemostasis Field: A Scoping Review

**DOI:** 10.1111/ijlh.14403

**Published:** 2024-12-10

**Authors:** H. Mansouritorghabeh, A. Monard, F. Heubel‐Moenen, J. Leentjens, A. Stroobants, Y. Henskens

**Affiliations:** ^1^ Central Diagnostic Laboratories, Ghaem Hospital Mashhad University of Medical Sciences Mashhad Iran; ^2^ Department of Internal Medicine, Division of Thrombosis and Hemostasis Einthoven Laboratory for Vascular and Regenerative Medicine, Leiden University Medical Center Leiden The Netherlands; ^3^ Department of Internal Medicine – Hematology Maastricht University Medical Centre+ Maastricht The Netherlands; ^4^ CARIM–School for Cardiovascular Disease Maastricht University Maastricht The Netherlands; ^5^ Department of Internal Medicine–Vascular Medicine Radboud University Medical Center, RadboudUMC Nijmegen The Netherlands; ^6^ Department of Clinical Chemistry Radboud University Medical Center, RadboudUMC Laboratory for Diagnostics and Laboratory for Hematology Nijmegen The Netherlands; ^7^ Central Diagnostic Laboratory, Unit for Hemostasis and Transfusion Maastricht University Medical Centre+ Maastricht The Netherlands

**Keywords:** anticoagulants, bleeding disorders, flow chamber, hemophilia, laboratory analysis, platelet, Total Thrombus‐Formation Analysis System (T‐TAS), von Willebrand disease, von Willebrand factor

## Abstract

**Background:**

A wide variety of laboratory hemostasis tests is available, but the majority is plasma‐based, static and unable to assess platelet function and fibrin formation simultaneously. The Total Thrombus‐Formation Analysis System (T‐TAS) is a microchip‐based flow chamber system that simulates in vivo conditions for evaluating whole blood thrombogenicity.

**Aim:**

A comprehensive overview of its applicability in different thrombosis and hemostasis related clinical situations is lacking and therefore this scoping review was performed.

**Materials & methods:**

A literature search was done using the electronic databases PubMed, Scopus and Embase on January 7, 2024. Original studies assessing the usefulness of the T‐TAS in thrombosis and hemostasis related clinical situations were eligible for this scoping review.

**Results:**

A total of 28 studies were included; six studies investigating the role of the T‐TAS in congenital bleeding disorders, five studies using the T‐TAS to assess 1‐year bleeding risk in patients on antiplatelet or anticoagulant medications, four studies investigating the effects of thrombocytopenia and hemodialysis on thrombus formation as measured by the T‐TAS, 11 studies testing the applicability of the T‐TAS in the monitoring of anticoagulant and antiplatelet therapies and eventually two studies on the ability of the T‐TAS to assess the thrombogenicity in different disease entities.

**Discussion & conclusion:**

The T‐TAS method is an interesting technology that mimics the complex biological coagulation process using shear forces, creating a “blood vessel component on a chip”. More research is needed, but it could eventually function as a screening test for platelet function and coagulation. Moreover, it could be used to detect the presence of anticoagulant and/or antiplatelet medication.

## 1 Introduction

Hemostasis is the process of blood clot formation at the site of vessel injury under arterial or venous flow. It is a complex process consisting of primary and secondary hemostasis and it is regulated by the fibrinolytic system to prevent excessive fibrin formation. Platelet adhesion, platelet activation and platelet aggregation are different mechanisms of primary hemostasis, leading to the initial platelet plug formation. Secondary hemostasis results in formation of thrombin. Activated platelets are able to bind coagulation factors to their procoagulant surface on which the coagulation cascade takes place, resulting in thrombin formation. The formation of thrombin eventually leads to the conversion of fibrinogen to fibrin. Fibrin fibers are able to form cross‐linked networks, resulting in a more stable clot [1]. Fibrinolysis is the process that degrades the formatted clots by converting fibrin into fibrin degradation products.

A wide variety of laboratory hemostasis tests is available to assess primary and secondary hemostasis. The majority of the tests is performed in plasma, but some (global) hemostasis assays are performed in whole blood. A distinction can be made between screening and confirmatory tests. The prothrombin time (PT) and activated partial thromboplastin time (aPTT) are plasma‐based tests used for screening of secondary hemostasis abnormalities in clinical practice, but their sensitivity and specificity is rather low [2, 3]. The platelet function analyzer (PFA‐200) is a whole‐blood test that is used for screening of primary hemostasis function (platelet function and VWD), using very high shear rates. It only provides closure times (CT) and normal PFA‐200 results do not exclude mild thrombocytopenia or VWD [4, 5]. Plasmatic tests for quantification of coagulation factor levels are used as confirmatory tests to diagnose congenital and acquired coagulation factor deficiencies and VWD [2, 6]. To confirm a platelet function defect, light transmission aggregometry (LTA), performed in platelet rich plasma, is generally considered as the gold standard [7, 8]. The main disadvantage of LTA is the labor‐intensiveness of the assay and its sensitivity to several pre‐analytical conditions [9]. Adenosine triphosphate (ATP) release is a test to assess platelet dense granule release [10]. Another test for primary hemostasis is the impedance aggregometry (Multiplate), which measures platelet aggregation in whole blood. This test can be useful for diagnosing platelet function disorders [11, 12], for monitoring antiplatelet therapy [13] and/or for predicting the bleeding risk in a perioperative setting [14, 15, 16] but lacks the sensitivity of LTA [17]. Viscoelastic tests such as ROTEM, TEG, ClotPro, Quantra and Sonoclot are whole blood tests introduced for rapid monitoring of plasmatic coagulation, fibrinolysis and platelet count in (massive) bleeding. Dedicated, point of care, whole blood platelet function tests for monitoring antiplatelet therapy such as VerifyNow have been evaluated frequently to detect or exclude the presence of aspirin or P2Y12 inhibitors [18].

Important limitations of the above mentioned tests are the fact that most tests are static, not involving any form of shear, and that they don't take interaction between primary and secondary hemostasis into account. In addition, for the platelet function tests, there is a wide range of assays with different modes of action but poor agreement between the tests [19]. Another issue with the currently available platelet function tests is the inability to provide information on platelet function in patients with thrombocytopenia and/or low hematocrit levels, for example in patients with chemotherapy induced thrombocytopenia and bleeding symptoms.

There is a strong need for hemostatic assays that are able to measure both platelet function and fibrin formation simultaneously, in whole blood and incorporating shear in the model, to better depict the in vivo thrombus formation and coagulation process, providing more accurate information by looking at the hemostatic system as a whole.

**FIGURE 1:**
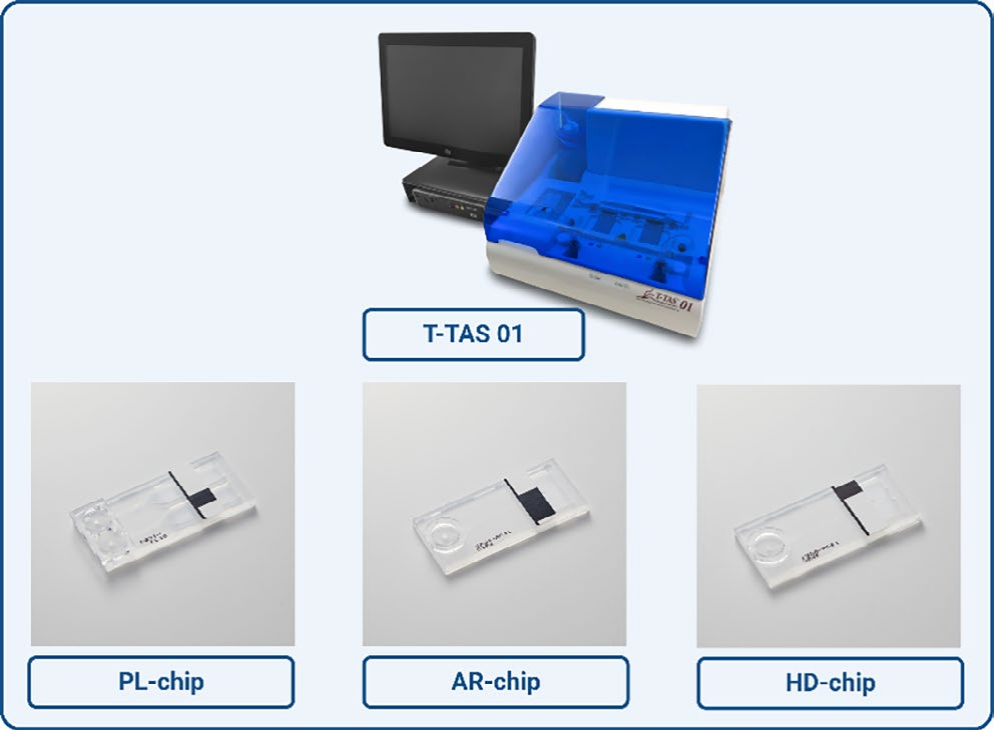
Picture of the T‐TAS 01 system and the three available chips.

The Total Thrombus‐Formation Analysis System (T‐TAS) (Zacros, Fujimori Kogyo Co. Ltd. Tokyo, Japan) is a microchip‐based flow chamber system introduced in 2011 by Hosokawa K. et al. [20] (Figure 1). This system operates under physiological shear rate and mimics in vivo thrombus formation by measuring platelet function and/or fibrin formation, depending on the chip used, as in physiological hemostasis.

The system is equipped with a pneumatic pump, a flow pressure pump and a rectangular capillary chip. It is an automatic analyzer and claims to provide an easy and rapid quantitative analysis of platelet aggregation, thrombus formation and/or fibrin formation in the microchip under shear flow. By continuous monitoring of variations in flow pressure in the microchip, the thrombus development process can be analyzed [21]. This system consists of three types of microchips (platelet chip (PL), atheroma chip (AR) and hemodilution chip (HD)) with different designs and functions that are further explained in Table 1.

**TABLE 1:** Characteristics of the AR‐, PL‐ and HD‐chips used in T‐TAS.

Cartridge	Needed anticoagulant(s)	Sample volume (μL)	Sample stability (for T‐TAS 01)	Capillary coated with	Size of capillary	Nr. of capillary channels	Shear rate (S‐1)	Type of thrombus Indication for use	Assay time
Atheroma (AR)‐chip	Citrate* or Hirudin**	450	30 min—3 h after blood collection	Type I collagen and tissue thromboplastin	300 μm wide × 80 μm deep	1	240 or 600	Designed for assessment of whole blood hemostasis, including primary and secondary hemostatic functions	~30 min
Platelet (PL)‐chip	Hirudin** BAPA***	320	30 min—6 h after blood collection	Type I collagen	40 μm wide × 40 μm deep	2	1000 1500 2000	Designed for quantitative analysis of platelet adhesion & aggregation, granule secretion and thrombus growth	~10 min
Hemodilution (HD)‐chip	Citrate	440–460	30 min—3 h after blood collection	Type I collagen and tissue thromboplastin	0.3 mm × 50 μm	1	1200	Designed for assessment of whole‐blood hemostasis (primary and secondary hemostasis) in samples with low platelet count	~30 min

*Note:* The flow rate in T‐TAS 01 is fixed and cannot be modified by the user's manually. In T‐TAS Plus, users can select flow rate from several options.

Abbreviations: AR; atheroma, Nr; number, Pl; platelet.

* 3.8% sodium citrate solution.

** The final concentration of Hirudin is 25 μg/m.

*** Benzylsulfonyl‐D‐Arg‐Pro‐4‐amidinobenzylamide, ~ about.

Approximately 320 μL of whole blood circulates through a capillary channel coated with activators processed by a dedicated computer. As a result, platelets adhere to the thrombogenic surface and form a platelet aggregate with (AR and HD chip) or without (PL) fibrin formation. The formed thrombus leads to an increase in endogenous pressure in the system until the capillaries are completely occluded. The rising pressure of blood and thrombus hardness in the chamber (PL, AR, or HD) reflects the potential of the patient's blood to form a thrombus. T‐TAS provides multiple outcome measures including occlusion start time (OST), occlusion time (OT), and area under the curve (AUC).

This scoping review attempts to provide a comprehensive overview of studies of the T‐TAS system in the field of thrombosis and hemostasis. In this context, the available information in the literature on T‐TAS and its advantages and disadvantages are reviewed.

## 2 Methods

### 2.1 Protocol

For this scoping review, the following research question was formulated: ‘What is known from the existing literature about the T‐TAS and its effectiveness in detecting bleeding and thrombotic disorders and the monitoring of anticoagulant therapy?’. The methodological quality of all eligible studies was assessed using the Joanna Briggs Institute (JBI) Appraisal tool. This scoping review was conducted according to the guidelines for scoping reviews by Hilary Arksey and Lisa O'Malley [22].

### 2.2 Literature Search

**FIGURE 2:**
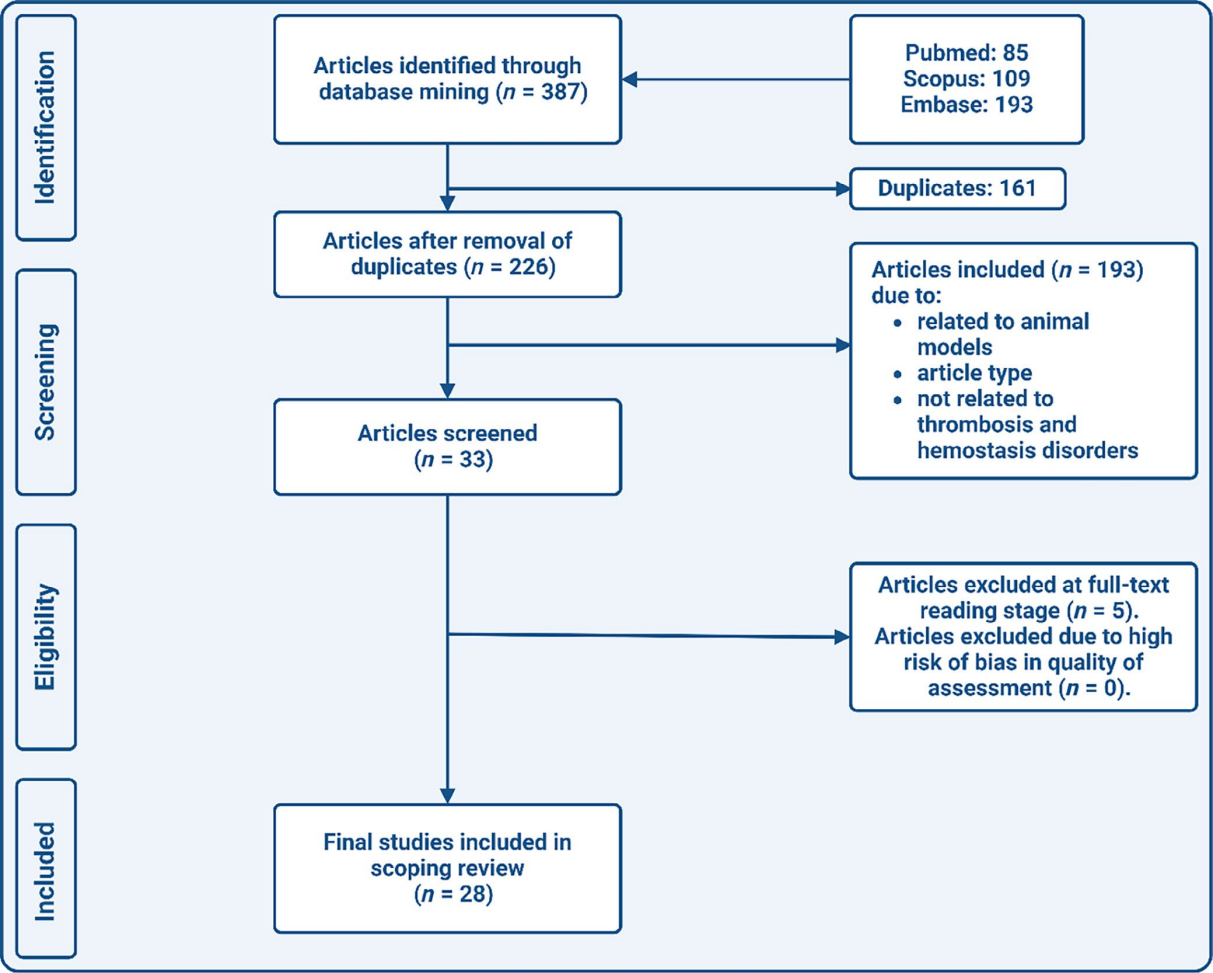
Flowchart of the study selection.

The literature search was performed using the electronic databases PubMed, Embase, and Scopus. The following search terms were used to identify relevant studies: “total thrombus formation analysis” OR “total thrombus analysis system” OR “T‐TAS”. The final search date was January 7, 2024. No restrictions concerning language or date were applied. Editorials, commentaries, case reports, reviews and poster or conference abstracts were excluded. All references from eligible studies were checked for other possible relevant titles, until no new titles were found. A flow‐diagram of the search strategy can be found in Figure 2.

### 2.3 Identification of Relevant Studies

Eligible studies for this scoping review were original articles investigating the usefulness of T‐TAS applied to patients with bleeding disorders, thrombotic disorders or patients using antiplatelet and/or anticoagulant therapy. In order to be included in this review, studies had to meet the following criteria: [1] T‐TAS experiments were performed on human samples, [2] study population was *n* ≥ 10 and [3] T‐TAS was used to investigate bleeding and thrombosis disorders or monitoring of anticoagulant/antiplatelet therapy. Papers that examined T‐TAS in animal models or the effects of medicinal herbs on coagulation were excluded. All titles and abstracts were independently screened by two reviewers (A.M. and H.M.) and the pre‐specified in‐ and exclusion criteria were applied during the process. The full text of potentially useful articles was then obtained. Disagreements on inclusion of articles were resolved by consensus.

### 2.4 Quality Assessment

Two reviewers (H. M. and A.M.) independently assessed the methodological quality of the included studies. The JBI Appraisal Tool (checklists) were used (Available from https://jbi.global/critical‐appraisal‐tools. Accessed on January 15, 2023). The checklist for case control studies, cohort studies, and diagnostic test accuracy studies was used, dependent on the study design. Each selected study was assigned a score from 0 to 10. The risk of bias was determined by summing the allocated scores. The final score of 7–10 was considered as low risk of bias, 4–6 was as moderate risk of bias, and 0–3 as high risk of bias. Each conflict was resolved in a joint meeting. The quality assessment checklists can be found in Table S1.

### 2.5 Data Extraction

Data was extracted from each included article by both reviewers (A.M. and H.M.) using a pre‐specified data extraction form (Table S2). The following information was extracted from each included study: First author and year of publication, title of the full text article, study location, study design, source of study population, sample size, targeted condition, used T‐TAS chips and shear rates, reference standard used (golden standard laboratory test), analysis used for this review, outcome measure and authors conclusion.

## 3 Results

### 3.1 Study Selection

The literature search in PubMed, Scopus and Embase resulted in 387 results. After deduplication, 226 articles remained. During the screening of titles and abstracts, 178 articles were excluded. From the remaining 48 potentially relevant studies, the full text version was obtained. Eventually, 28 studies were included in this scoping review (Figure 2).

### 3.2 Study Characteristics

The study characteristics of the 28 included studies are summarized in Tables S3–S6. Six studies investigated the capability of T‐TAS in diagnosing congenital bleeding disorders and monitoring their treatment (Table S3). A total of 11 articles focused on the usefulness of T‐TAS in monitoring patients with anticoagulant and/or antiplatelet therapy (Table S4). Five studies assessed the ability of T‐TAS in predicting the bleeding risk in different study populations (Table S5). Eventually, Two studies used T‐TAS to assess thrombogenicity in different disease entities (Table S6). One article investigated the effect of thrombocytopenia on T‐TAS parameters and three studies applied T‐TAS before and after hemodialysis (Table S6).

### 3.3 Methodological Quality Assessment

No studies with a high risk of bias were identified. Nineteen studies were determined to have a low risk of bias, the remaining nine studies were determined to have a moderate risk of bias. No studies were excluded based on the quality assessment.

### 3.4 The Role of T‐TAS in the Diagnosis and Treatment of VWD and Platelet Function Disorders (PFD) (Table S3)

Six studies investigated the role of T‐TAS in the diagnosis and treatment of patients with VWD and PFD. A short summary is given for each study and detailed study characteristics and outcomes can be found in Table S3. The study by Daidone et al. showed that T‐TAS is able to detect patients with severe Type 1 VWD and patients with Type 2A and 2B VWD with abnormal multimers. Patients with mild Type 1 VWD and type 2 M VWD were not detected.

Nogami et al. investigated the usefulness of T‐TAS in predicting the bleeding phenotype in Type 1 VWD patients [24]. There was a weak to moderate correlation between T‐TAS parameters, bleeding score and VWF:Ag levels.

Oggiwara et al. observed abnormal T‐TAS results in one pediatric Type 1 VWD patient, one adult Type 2A VWD, one adult Type 2 N VWD patient, and one pediatric Type 3 VWD [25].

A study by Ägren et al. showed reduced thrombin formation in Type 3 VWD patients, which improved significantly after treatment with VWF‐FVIII concentrate [26].

Lecchi et al. included patients with suspected PFD and unexplained mild to moderate bleeding [27] in whom T‐TAS did not show significant differences in median parameters compared to healthy controls.

Lastly, Nakajima et al. showed that T‐TAS is able to detect Type 2A, 2B and 3 VWD and severe PFDs [28].

**FIGURE 3:**
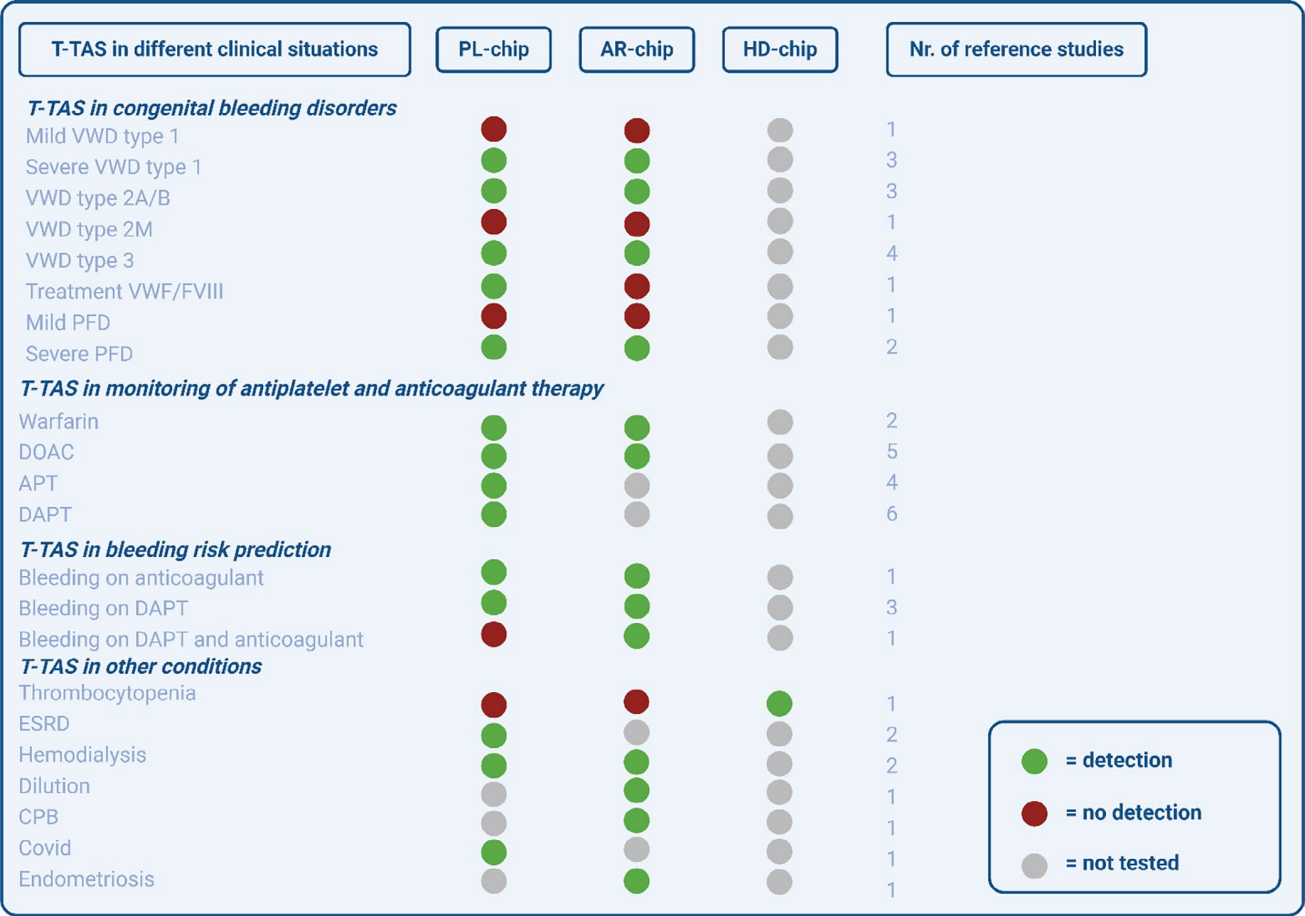
Overview of the ability of the T‐TAS to detect abnormalities in different clinical situations.

In conclusion, T‐TAS is able to discriminate severe Type 1 VWD, VWD Type 2A, 2B and 3, but not mild VWD Type 1 and VWD 2 M. T‐TAS is also able to detect severe PFD, but not milder forms of PFD. Overall, the PL‐chip seems to be more sensitive for VWF‐mediated platelet function than the AR‐chip (Figure 3).

### 3.5 The Role of T‐TAS in Monitoring Anticoagulant and Antiplatelet Therapies (Table S4)

Eleven studies investigated the role of T‐TAS in monitoring of anticoagulant and antiplatelet therapies. A short summary is given for each study and detailed study characteristics and outcomes can be found in Table S4. Idemoto et al. used T‐TAS to investigate the antithrombotic effects of DOACs and showed that T‐TAS is able to detect dabigatran and apixaban, but not rivaroxaban [29].

Three studies on patients with atrial fibrillation (AF) were performed using DOAC's [30, 31, 32]. Ishii et al. observed significantly different T‐TAS parameters in patients taking anticoagulants compared to patients not taking anticoagulants. Sugihara et al. performed T‐TAS measurements at DOAC through and peak levels and showed increasingly abnormal T‐TAS results at peak levels. Taune et al. evaluated thrombus formation by T‐TAS and ROTEM at trough and peak levels of dabigatran [32]. T‐TAS parameters correlated significantly with dabigatran concentrations, as well as ROTEM clotting time (CT).

Sueta et al. showed that T‐TAS is able to detect edoxaban treatment by measuring before and 7 days after the start of edoxaban treatment [33].

The study by Arima et al. performed T‐TAS measurements in patients receiving (dual) antiplatelet therapy (D)APT and showed significantly different results compared to healthy controls [34]. This was similar to the results measured by VerifyNow.

Zheng et al. also investigated the role of T‐TAS in patients using DAPT and showed abnormal results, significantly lower than the cutoff value [35].

Lecchi et al. analyzed residual platelet function in 26 CAD‐ patients treated with (D)APT using T‐TAS and comparing T‐TAS results with LTA results, showing a test agreement of 54% [27].

A study by Borst et al. used T‐TAS and TG to investigate the effects of DAPT with or without low dose rivaroxaban in patients with acute coronary syndrome (ACS) [36] and showed that both T‐TAS and TG provide significantly different results after initiation of rivaroxaban treatment.

Matsuo et al. performed T‐TAS measurements in 20 pediatric Fontan patients, with and without antithrombotic therapy (aspirin and/or warfarin) and showed significantly different results compared to a control group [37].

Lastly, Kikuchi et al. used T‐TAS and VerifyNow in patients with ST‐elevation myocardial infarction undergoing primary percutaneous coronary intervention (PPCI) and treated with DAPT and unfractionated heparin (UFH) [38]. Results showed that T‐TAS and VerifyNow levels decreased over time.

In conclusion, multiple studies observed lower AR‐AUC levels in patients using DOACs, whilst PT and aPTT often showed no abnormalities in patients using DOACs [39]. T‐TAS could thus serve as a screening test for DOAC‐use, but there was only a weak correlation between DOAC‐concentration and T‐TAS results. The T‐TAS PL‐chip is able to detect APT and DAPT and results were comparable to those of the VerifyNow (Figure 3).

### 3.6 The Role of T‐TAS in the Prediction of Bleeding Events in Patients on Anticoagulant and/or Antiplatelet Therapy (Table S5)

Five studies investigated the role of T‐TAS in predicting bleeding events in patients on anticoagulant and/or antiplatelet treatment. A short summary is given for each study and detailed study characteristics and outcomes can be found in Table S5. Ito et al. investigated the role of T‐TAS in predicting periprocedural bleeding events in AF‐patients treated with warfarin or DOACs and undergoing catheter ablation (CA) [40]. Results showed significantly different T‐TAS results in patients with bleeding complications compared to those without, whilst PT and aPTT did not differ significantly.

Nakanishi et al. performed T‐TAS in patients treated with DAPT undergoing PCI and showed significantly different T‐TAS parameters in the high bleeding risk group [41].

The study by Oimatsu et al. investigated the role of T‐TAS in predicting periprocedural bleeding events in coronary artery disease (CAD) patients treated with DAPT and undergoing PCI and showed significantly different results in patients with bleeding complications compared to those without [42].

Ichikawa et al. used T‐TAS to investigate bleeding complications in CAD‐patients using oral anticoagulation (DOAC or vitamin K antagonist) combined with DAPT [21]. T‐TAS results were significantly different in the bleeding complication group. Lastly, Mitsuse et al. used the T‐TAS to predict the 1‐year bleeding risk in CAD‐patients treated with DAPT and/or DOACs and showed significantly different T‐TAS parameters in bleeding patients compared to nonbleeding patients [43].

In conclusion, the T‐TAS AR‐chip was able to predict bleeding in CAD‐patients on oral anticoagulation and in AF‐patients undergoing CA (Figure 3).

### 3.7 The Role of T‐TAS in Other Diseases and Conditions (Table S6)

Six studies investigated the role of T‐TAS in other diseases and conditions. A short summary is given for each study and detailed study characteristics and outcomes can be found in Table S6. Atari et al. performed T‐TAS measurements in thrombocytopenic ICU‐patients, before and after platelet transfusion and showed that the HD‐chip is able to detect recovery of the hemostatic function after platelet transfusion in these patients [44].

In the study by Mitic et al. T‐TAS was used in end‐stage renal disease (ESRD) patients, before and after hemodialysis [45]. Impaired T‐TAS results were seen in these patients compared to healthy controls, both before and after hemodialysis.

The study by Nakanishi et al. investigated the influence of hemodialysis on thrombogenicity in patients undergoing PCI and using DAPT using T‐TAS and showed impaired T‐TAS parameters in patients undergoing hemodialysis compared to those not on hemodialysis [46]. Moreover, T‐TAS was able to distinguish between bleeding and nonbleeding patients.

Ogawa et al. looked at haemodilution induced changes in coagulation before and after cardiopulmonary bypass (CPB) [47]. Results showed prolonged T‐TAS parameters after CPB compared to baseline.

Ghirardello et al. investigated platelet thrombus formation in COVID‐19 patients [48] and showed significantly different T‐TAS results compared to a control group, indicating impaired thrombus formation in COVID‐19.

Lastly, one study by Kedzia et al. performed T‐TAS measurements in patients with endometriosis and showed a prothrombotic state in these patients compared to healthy controls [49].

In conclusion, the HD‐chip seems to be able to discriminate between thrombocytopenic patients with and without a bleeding tendency. Mixed results were seen in hemodialysis patients. In COVID‐19, T‐TAS Pl‐chip showed lower thrombogenicity and in endometriosis, T‐TAS AR‐chip showed higher thrombogenicity (Figure 3).

## 4 Discussion

This review aimed to provide a comprehensive overview of the utility of the T‐TAS system in the field of thrombosis and hemostasis.

Our review shows that in VWD and PFD‐patients, T‐TAS seems to be able to only detect patients with severe primary hemostasis defects, and not milder forms of VWD and/or PFD. The PL‐chip is able to discriminate VWD Type 2A, 2B and 3, but not VWD Type 1 and VWD Type 2 M [23, 25]. Similar results were seen in studies evaluating the PFA in VWD patients, showing that the PFA is mainly able to detect severe VWD types and abnormal VWF multimer patterns [50, 51].

The results regarding the detection of PFD are similar to results from studies showing that the PFA and multiplate are able to discriminate severe PFD's, but not mild PFD's [17]. This indicates that for the diagnosis of PFD, LTA remains the golden standard.

T‐TAS could be of added value in monitoring patients receiving VWF‐FVIII treatment as one study suggests that the AR‐chip was able to assess the differences in coagulation‐dependent thrombus formation in VWD Type 3 patients, before and after administration of VWF‐FVIII treatment. This is not possible with the PFA, as a previous study showed that PFA normalizes after DDAVP treatment, but not always after VWF‐FVIII treatment [52].

Several included studies investigated different types of anticoagulant and antiplatelet therapies. The overall conclusion is that T‐TAS is able to detect the presence of warfarin, DOAC's, APT and DAPT. Several studies observed lower AR‐AUC levels in patients using DOAC's, whilst PT and aPTT often showed no abnormalities. This is in concordance with previous research, showing that the detection of dabigatran and rivaroxaban is difficult with aPTT and PT, but is possible, dependent on the reagents that are used [39]. The correlation between DOAC concentrations and T‐TAS results was investigated in only one study, showing weak but significant correlations between DOAC concentrations and T‐TAS results [32].

Studies on APT and DAPT showed that this can be detected by the T‐TAS PL‐chip. Comparable results were seen between T‐TAS and VerifyNow in discriminating patients using DAPT [34, 38]. One possible advantage of the T‐TAS is its overall assessment of platelet function, whilst the VerifyNow system is designed to detect certain platelet activation routes in particular [53].

Several studies looked at bleeding risk prediction in different populations. In CAD‐patients on oral anticoagulation, T‐TAS was able to predict subsequent bleeding events and in AF‐patients undergoing CA, T‐TAS was able to predict periprocedural bleeding events. These studies were performed in rather large study populations. In general, the AR‐chip seems to be a better predictor of bleeding than the PL‐chip.

An important advantage of T‐TAS is its ability to assess platelet function in thrombocytopenic patients, using the HD‐chip. This could be relevant for patients with chemotherapy induced thrombocytopenia amongst others. Up until today, these patients receive platelet transfusions based on platelet count, and not platelet function [54]. Moreover, some of these patients bleed despite platelet transfusions whilst others don't bleed at all. It would therefore be relevant to have other, more accurate parameters to base transfusion decisions on [55]. One study showed that T‐TAS was able to discriminate between thrombocytopenic patients with a bleeding tendency and those without and to detect recovery of hemostasis after platelet transfusion [56].

In COVID‐19 patients, reduced platelet thrombogenicity measured by T‐TAS was seen compared to controls in one study [48]. These results are in contrast to other studies that show increased platelet activity and reactivity in COVID‐19 patients [57]. In patients with endometriosis, T‐TAS results showed a prothrombotic state [49]. This is in accordance with one other study describing increased TG‐parameters [58].

Based on the results from the included studies in this review, the T‐TAS system seems to be a promising tool in different clinical situations. However, there are still some (major) limitations and knowledge gaps. First of all, there is a wide variety of T‐TAS outcome parameters, leading to difficulties with interpretation, but also generalizability of the results as it is challenging to compare results from different studies when different outcome measures were used. There is now also still a lack of well‐defined reference values. In Table S7, a table with reference values from all the included studies can be found. This problem could be solved in future research by either providing all the possible T‐TAS parameters or by agreeing on a set of important parameters. Future research should also investigate the clinical value of each of these parameters.

Second, the majority of the included studies in this review used rather small study populations. Hence, most of the experiments need further investigation in studies with a larger sample size. Moreover, heterogeneous patient populations were investigated, especially in the studies investigating the use of anticoagulants and/or DAPT and the studies on hemodilution. This leads to difficulty in comparing these studies with each other. Therefore, it is hard to draw generalizable conclusions. In the studies investigating the use of DOAC's, the correlation of the T‐TAS results with plasma concentrations of medication should be further investigated, as this is major limitation of the current studies.

An important limitation of the currently available literature on T‐TAS is the lack of comparison of T‐TAS with the current golden standard for platelet function testing, the LTA. Only a few studies included in this review compared T‐TAS results with LTA results, but the majority of the studies didn't. Therefore, it is difficult to draw any conclusions regarding the added value of T‐TAS on top of the currently available diagnostic tests. Future research on T‐TAS should also include LTA results to further investigate this and whether T‐TAS could increase the diagnostic yield, for example in patients with bleeding disorder of unknown cause.

Another limitation of T‐ TAS is the lack of integration of the endothelial function. Endothelial cells regulate platelets, coagulation, and fibrinolysis function in vivo. Although some efforts have been made to recruit vascular cells and incorporate them in the hemostasis assay, they have not been successful to date. Another challenge in this regard is that the phenotype of endothelial cells varies in different organs [59].

The clinical applicability of T‐TAS is also still limited, as it is now mainly used in research settings. There is only a limited number of studies on T‐TAS in the field of thrombosis and hemostasis, which is due to the fact that it is a newly manufactured product. Interpretation of the tests results will require attention as clinicians will have to be educated. Based on the currently available studies, no strong conclusions concerning the clinical added value of the T‐TAS can be drawn yet.

## 5 Conclusion

Despite these limitations, the T‐TAS method is an interesting technology that mimics the complex biological coagulation process using shear force. More research is needed, but it could eventually serve as a screening test for platelet function and coagulation. Moreover, it could be used to detect the presence of anticoagulant and/or antiplatelet medication.

## Author Contributions

**H. Mansouritorghabeh:** first draft, literature search, study selection, quality check, and writing. **A. Monard:** literature search, study selection, quality check, writing, tables and figures. **F. Heubel‐Moenen:** review and editing, supervision. **J. Leentjens:** review and editing, supervision. **K. Stroobants An:** review and editing, supervision. **Y. Henskens:** review and editing, supervision.

## Ethics Statement

The authors have nothing to report.

## Consent

The authors have nothing to report.

## Conflicts of Interest

The authors declare no conflicts of interest.

## Supporting information


**Table S1** Overview of the quality assessment forms.
**Table S2**: Data extraction form.
**Table S3**: Characteristic and finding of studies that have investigated T‐TAS efficacy in congenital bleeding disorders.
**Table S4**: Characteristic features of studies that investigated the monitoring of anticoagulant and antiplatelet therapies by T‐TAS.
**Table S5**: Characteristic features of studies that investigated the role of T‐TAS in bleeding risk prediction.
**Table S6**: Characteristic features of studies that investigated role of hemodialysis and thrombocytopenia on T‐TAS markers and thrombogenecity in COVID‐19 and endometriosis.
**Table S7**: Reference ranges measured in healthy volunteers in different study groups.

## Data Availability

The data that support the findings of this study are available from the corresponding author upon reasonable request.
