# Supplementary material for: The Utility of Total Thrombus‐Formation Analysis System (T‐TAS) in the Thrombosis and Hemostasis Field: A Scoping Review
Source: Int J Lab Hematol. 2024 Dec 10;47(2):201–11. doi: 10.1111/ijlh.14403 (PMC11885686; doi:10.1111/ijlh.14403)
Supplement: Supplementary file 1 — Table S1 Overview of the quality assessment forms. Table S2: Data extraction form. Table S3: Characteristic and finding of studies that have investigated T‐TAS efficacy in congenital bleeding disorders. Table S4: Characteristic features of studies that investigated the monitoring of anticoagulant and antiplatelet therapies by T‐TAS. Table S5: Characteristic features of studies that investigated the role of T‐TAS in bleeding risk prediction. Table S6: Characteristic features of studies that investigated role of hemodialysis and thrombocytopenia on T‐TAS markers and thrombogenecity in COVID‐19 and endometriosis. Table S7: Reference ranges measured in healthy volunteers in different study groups. [file IJLH-47-201-s001.docx]

**Supplementary materials**

Table S1: Overview of the quality assessment forms

| **Study** | **Quality assessment tool** | **Record number** |
| --- | --- | --- |
| Agren at al. 2017 | JBI critical appraisal checklist for diagnostic test accuracy studies | 7 |
| Daidone et al. 2016 | JBI critical appraisal checklist for diagnostic test accuracy studies | 7 |
| Lecchi et al. 2023 | JBI critical appraisal checklist for diagnostic test accuracy studies | 8 |
| Nakajima et al. 2021 | JBI critical appraisal checklist for diagnostic test accuracy studies | 6 |
| Nogami et al. 2016 | JBI critical appraisal checklist for diagnostic test accuracy studies | 6 |
| Ogiwara et al. 2015 | JBI critical appraisal checklist for diagnostic test accuracy studies | 5 |
| Ichikawa et al. 2019 | JBI critical appraisal checklist for cohort studies | 8 |
| Ito et al.2016 | JBI critical appraisal checklist for cohort studies | 7 |
| Mitsuse et al. 2020 | JBI critical appraisal checklist for cohort studies | 7 |
| Nakanishi et al. 2021 (PCI) | JBI critical appraisal checklist for cohort studies | 8 |
| Oimatsu et al. 2017 | JBI critical appraisal checklist for cohort studies | 6 |
| Atari et al. 2020 | JBI critical appraisal checklist for cohort studies | 6 |
| Mitic et al.2022 | JBI critical appraisal checklist for cohort studies | 5 |
| Nakanishi et al. 2021 (HD) | JBI critical appraisal checklist for cohort studies | 8 |
| Ogawa et al. 2013 | JBI critical appraisal checklist for case control studies | 5 |
| Arima et al. 2016 | JBI critical appraisal checklist for diagnostic test accuracy studies | 8 |
| Zheng et al. 2022 | JBI critical appraisal checklist for diagnostic test accuracy studies | 4 (no reference test was used) |
| Borst et al. 2017 | JBI critical appraisal checklist for cohort studies | 8 |
| Idemoto et al. 2017 | JBI critical appraisal checklist for diagnostic test accuracy studies | 6 |
| Ishii et al. 2017 | JBI critical appraisal checklist for diagnostic test accuracy studies | 8 |
| Matsuo et al. 2022 | JBI critical appraisal checklist for case control studies | 8 |
| Sueta et al. 2015 | JBI critical appraisal checklist for diagnostic test accuracy studies | 7 |
| Sugihara et al. 2016 | JBI critical appraisal checklist for diagnostic test accuracy studies | 8 |
| Taune et al. 2017 | JBI critical appraisal checklist for diagnostic test accuracy studies | 7 |
| Kikuchi et al. 2020 | JBI critical appraisal checklist for diagnostic test accuracy studies | 7 |
| Ghirardello et al, 2021 | JBI critical appraisal checklist for cohort studies | 8 |
| Kedzia et al. 2023 | JBI critical appraisal checklist for case control studies | 8 |

| **First author, year** |  |
| --- | --- |
| **Title** |  |
| **Study location** |  |
| **Study design** |  |
| **Source of study population** |  |
| **Sample size** |  |
| **Targeted condition** |  |
| **Used chip and shear rate** |  |
| **Reference standard** |  |
| **Analysis used for review** |  |
| **Outcome measure(s)** |  |
| **Authors conclusion** |  |

Table S2: Data extraction form

Table S3: Characteristic and finding of studies that have investigated T-TAS efficacy in congenital bleeding disorders.

| **First author, year** | **Title** | **Study location** | **Study design** | **Source of study population** | **Sample size** | **Targeted condition** | **Used chip and shear rate** | **Reference standard** | **Analysis used for review** | **Outcome measure(s)** | **Authors conclusion** |
| --- | --- | --- | --- | --- | --- | --- | --- | --- | --- | --- | --- |
| **Daidone et al, 2016** | Usefulness of the Total Thrombus-Formation Analysis  System (T-TAS) in the diagnosis and characterization of von Willebrand disease | Italy | Case control | (1)VWD patients  (2)healthy controls | (1)30  (2)20 | The identification and characterization of VWD-patients | PL-chip at 1167 $s^{-1}$  AR-chip at 300$s^{-1}$ | Conventional coagulation assays, VWF assays | OST, OT and AUC on PL- and AR-chip in patients with VWD type 1, 2A, 2B and Vicenza | OST  OT  AUC | T-TAS seems to be sensitive to plasma VWF concentration and the presence of large multimers. |
| **Nogami et al, 2016** | Assessing the clinical severity of type 1 von Willebrand  disease patients with a microchip flow-chamber system | Japan | Case control | (1) VWD type 1 patients  (2)healthy controls | (1)50  (2)30 | Evaluation of the relationship between T-TAS, bleeding score (BS) and laboratory test results in VWD type 1 in order to evaluate and predict the BS in VWD type 1 by use of T-TAS | PL-chip at 1000 and 2000 $s^{-1}$  AR-chip at 240 $s^{-1}$ | Conventional coagulation assays, VWF assays | Correlation of T-TAS parameters with BS and VWF laboratory test results in VWD type 1 patients | PL-$T_{10}$  AR-$T_{10}$ | PL-$T_{10}$ correlated with BS and VWF:RCo levels and the Pl-chip was more sensitive than the AR-chip for VWF-mediated platelet function. |
| **Ogiwara et al, 2015** | Comprehensive evaluation of haemostatic function in von  Willebrand disease patients using a microchip-based flow  chamber system | Japan | Case control | (1)VWD patients  (2)healthy controls | (1)5  (2)20 | (a)Evaluation of thrombus formation in VWD patients before and after various treatments  (b)association between thrombogenicity and bleeding score (BS) | PL-chip at 1000 $s^{-1}$  AR-chip at 240 $s^{-1}$ | Conventional coagulation, VWF assays, ROTEM | Evaluation of thrombus formation with T-TAS in in vitro and in vivo | PL-$T_{10}$  PL-$T_{30}$  PL-$\mathrm{AUC}_{10}$  AR-$T_{10}$  AR-OT  AR-$\mathrm{AUC}_{30}$ | Platelet thrombus formation in the PL-chip is highly sensitive for VWF-mediated platelet function. Fibrin-rich platelet thrombus formation in the AR-chip is relatively insensitive for quantitative and qualitative VWF defects.  Combined PL- and AR-chips may be useful for the diagnosis of VWD. |
| **Ägren et al, 2017** | Monitoring of coagulation factor therapy in patients with von Willebrand disease type 3 using a microchip flow chamber system | Sweden | Case control | (1)VWD type 3 patients  (2)healthy controls | (1) 10  (2) 10 | Monitoring VWF-FVIII concentrate in VWD type 3 | PL-chip at 1000 $s^{-1}$  AR-chip at 240 $s^{-1}$ | Conventional coagulation assays, VWF assays | T-TAS measurements before and after VWF-FVIII treatment in VWD type 3 patients vs. healthy controls | $T_{10}$AR  $\mathrm{OT}_{80}$AR  $\mathrm{AUC}_{30}$AR  $\mathrm{AUC}_{10}$PL | Coagulation dependent thrombus formation (AR-chip) improved after VWF-FVIII treatment in VWD type 3 patients, effect on platelet dependent thrombus formation (PL-chip) was limited. |
| **Lecchi et al, 2023** | Flow-chamber device (T-TAS)  to diagnose patients suspected  of platelet function defects | Italy | Cohort | (1)patients referred to the outpatient clinic with mild to moderate bleeding tendency  (2)healthy controls | (1)96  (2)50 | The efficacy of T-TAS to detect congenital or acquired platelet function disorders in patients with unexplained bleeding | PL-chip at 2000 $s^{-1}$ | Conventional coagulation assays,  LTA | Efficay of T-TAS to detect PFD compared to LTA in patients with a mild-to-moderate bleeding tendency | OST  OT  AUC | T-TAS was able to identify patients with severe PFD (δ-SPD). Milder forms of PFD were not detected with T-TAS. |
| **Nakajima et al, 2021** | A microchip flow-chamber assay screens congenital primary  hemostasis disorders | Japan | Cohort | (1)VWD patients  (2)PFD patients | (1)22  (2)4 | The applicability of T-TAS as a screening tool for patients with suspected congenital primary hemostasis disorders | PL-chip at 1000 $s^{-1}$  AR-chip at 240 $s^{-1}$ | Conventional coagulation assays, flow cytometry, VWF multimer analysis, Multiplate | Ability of T-TAS to identify congenital primary hemostasis disorders | PL-$T_{10}$  AR-$T_{10}$ | The PL-chip distinguished all patients from healthy controls and could be a screening tool. The AR-chip distinguished VWD type 2B and 3 patients from healthy controls. PL-chip distinguished BSS and GT patients from healthy controls. |

AUC: area under curve, OT: occlusion time, OST: occlusion start time, PL; platelet, AR: atheroma, FVIII: factor VIII, VWF: von Willebrand factor, T-TAS: total thrombus formation system, LTA: light transmission aggregometry, ROTEM; Rotational thromboelastometry, δ-SPD: *Delta*-storage pool diseases, BSS: Bernard-Soulier syndrome, إBS: bleeding score, VWF:RCO; von Willebrand factor ristocetin cofactor, GT: glanzmann's thrombasthenia, PFD: platelet function diseases, VWF:Ag: von Willebrand antigen, S: second, S-1: per second.

Table S4: Characteristic features of studies that investigated the monitoring of anticoagulant and antiplatelet therapies by T-TAS.

| **First author, year** | **Title** | **Study location** | **Study design** | **Source of study population** | **Sample size** | **Targeted condition** | **Used chip and shear rate** | **Reference standard** | **Analysis used for review** | **Outcome measure(s)** | **Authors conclusion** |
| --- | --- | --- | --- | --- | --- | --- | --- | --- | --- | --- | --- |
| Idemoto et al, 2017 | Evaluation of the antithrombotic abilities of non‑vitamin  K antagonist oral anticoagulants using the Total  Thrombus‑formation Analysis System | Japan | Cohort | Hospitalized CVD patients  (1) with DOAC treatment  and  (2) without DOAC treatment | (1)78  (2)25 | Assessment of antithrombitic abilities of various NOAC’s in patients with CVD | PL-chip at 2000 $s^{-1}$  AR-chip at 600 $s^{-1}$ | Conventional coagulation assays | Assessment of thrombogenicity in CVD patients using different DOAC’s compared to CVD patients without DOAC | PL-AUC  AR-AUC | AR-AUC was significantly decreased in the DOAC-treated patient group compared to patients without DOAC treatment. Also significant decreases in PL-AUC were seen in DOAC-treated patients. |
| Ishii et al, 2017 | Direct Oral Anticoagulants Form  Thrombus Different From Warfarin  in a Microchip Flow Chamber  System | Japan | Cohort | AF patients who underwent RFCA and were treated with  (1)warfarin  (2)dabigatran  (3)rivaroxaban  (4)apixaban | (1)29  (2)19  (3)47  (4)25 | Assessment of differences in anticoagulant patterns of warfarin and DOAC’s in patients with AF who had undergone RFCA | AR-chip at 600 $s^{-1}$ | Conventional coagulation assays | Comparison of thrombogenicity before and 1 month after start treatment with DOAC or warfarin | $\mathrm{AR}_{10}$-$\mathrm{AUC}_{30}$ | $\mathrm{AR}_{10}$-$\mathrm{AUC}_{30}$ could be a marker for monitoring anticoagulant effects of warfarin and DOACS, but correlation with plasma DOAC concentration was weak. |
| Sueta et al, 2015 | A novel quantitative assessment of whole blood thrombogenicity in  patients treated with a non-vitamin K oral anticoagulant | Japan | Cohort | (1)patients undergoing total knee arthroplasty (TKA) and treated with edoxaban | (1)20 | Evalution of thrombogenicity in patients undergoing TKA before and after start edoxaban | PL-chip at 2000 $s^{-1}$  AR-chip at 600 $s^{-1}$ | Conventional coagulation assays | Effect of edoxaban on thrombogenecity measured with T-TAS | $\mathrm{PL}_{18}$-$\mathrm{AUC}_{10}$  $\mathrm{AR}_{10}$-$\mathrm{AUC}_{30}$ | $\mathrm{AR}_{10}$-$\mathrm{AUC}_{30}$ levels were significantly decreased 6 days after treatment with edoxaban, while $\mathrm{PL}_{18}$-$\mathrm{AUC}_{10}$  levels were almost identical before and after edoxaban treatment. |
| Sugihara et al, 2016 | Evaluation of the Antithrombotic Effects of Rivaroxaban and  Apixaban Using the Total Thrombus-Formation Analysis  System®: In Vitro and Ex Vivo Studies | Japan | Case control | (1)hospitalized AF-patients treated with rivaroxaban or apixaban  (2)healthy controls | (1)16  (2)20 | Evalution of the usefullness of T-TAS in monitoring the anticoagulant effects of NOAC’s | PL-chip  AR-chip | Conventional coagulation assays | Effect of DOAC’s on thrombogenicity measured by T-TAS | PL-AUC  AR-AUC | AR-AUC levels were significantly decreased in patients treated with rivaroxaban or apixaban. Anticoagulant effects of rivaroxaban and apixaban could be monitored with T-TAS. |
| Taune et al, 2017 | Whole blood coagulation assays ROTEM and T-TAS to monitor  dabigatran treatment | Sweden | Cohort | (1)AF-patients treated with dabigatran | (1)30 | Evaluation of thrombus formation in AF-patients treated with dabigatran using T-TAS and ROTEM and correlation with dabigatran concentrations | AR –chip at 600 $s^{-1}$ | Conventional coagulation assays, ROTEM | Correlation of T-TAS values to dabigatran concentrations in AF-patients compared to ROTEM | $T_{10}$  OT  AUC | T-TAS is able to detect differences in hemostasis during the dosing interval of dabigatran, but correlations with dabigatran plasma concentrations are weaker compared to ROTEM EXTEM and FIBTEM CT values. |
| Arima et al, 2016 | Assessment of platelet-derived thrombogenicity with the total thrombus formation analysis system in coronary artery disease patients recieving antiplatelet therapy | Japan | Cohort | Patients submitted to the cardiovascular department  (1)using no antiplatelet therapy  (2)using aspirin only  (3)using aspirin and clopidogrel | (1)56  (2)69  (3)149 | The assessment of effects of different antiplatelet therapies | PL-chip at 1500 $s^{-1}$ and 2000 $s^{-1}$  AR-chip at 600 $s^{-1}$ | VerifyNow P2Y12 assay | Assessment of thrombogenicity in patients using different kinds of antiplatelet therapies compared to controls | $\mathrm{PL}_{24}$-$\mathrm{AUC}_{10}$  $\mathrm{AR}_{10}$-$\mathrm{AUC}_{30}$ | $\mathrm{PL}_{24}$-$\mathrm{AUC}_{10}$ accurately assessed the therapeutic effect of antiplatelet therapy. |
| Zheng et al, 2022 | The Total Thrombus Formation (T-TAS) platelet  (PL) assay, a novel test that evaluates whole blood  platelet thrombus formation under physiological  conditions | The Netherlands, Hungary and Sweden | Cohort | CAD-patients undergoing PCI using DAPT  (1)clopidogrel + aspirin  (2)prasurgel + aspirin  (3)ticagrelor + aspirin | (1)22  (2)15  (3)20 | Evaluation of platelet function in CAD-patients using DAPT | PL-chip at 1500 $s^{-1}$ | NR | The ability of T-TAS to discriminate DAPT-treated patients from untreated patients | PL $\mathrm{AUC}_{10}$ | Compared to 260 healthy volunteers, all DAPT-treated patients had lower $\mathrm{AUC}_{10}$ values, suggesting excellent discrimination of DAPT-treated patients from untreated patients. |
| Lecchi et al, 2023 | Flow-chamber device (T-TAS)  to diagnose patients suspected  of platelet function defects | Italy | Cohort | (1)CAD patients treated with antiplatelet therapy  (2)healthy controls | (1)26  (2)50 | Evaluation of the ability of T-TAS to monitor the effects of antiplatelet therapy in CAD-patients | PL-chip at 2000 $s^{-1}$ | Conventional coagulation assays, LTA | The ability of T-TAS to assess residual platelet activity in patients on antiplatelet therapy compared to LTA | OST  OT  AUC | Test agreement on LTA vs. T-TAS concerning antiplatelet therapy responders was 54% (K CHOEN 0.150). |
| Borst et al, 2017 | Inhibitory mechanisms of very low–dose rivaroxaban in non–ST-elevation  myocardial infarction | Germany | Cohort | (1) NSTEMI patients treated with dual antiplatelet therapy (DAPT) and in vitro spiked with very low dose rivaroxaban | (1)40 | Coagulation- and platelet-dependent thrombus formation in patients treated with DAPT and the effect of in vitro addition of rivaroxaban | PL-chip at 1500 $s^{-1}$  AR-chip at 600 $s^{-1}$ | Thrombin generation, LTA | OST, OT and AUC on PL- and AR-chip in DAPT-treated patients and in vitro effect of additional spiking with rivaroxaban | OST  OT  AUC | Additional spiking of rivaroxaban resulted in significantly reduced thrombus formation in the AR-chip. Thrombus formation on the PL-chip was not affected. |
| Matsuo et al, 2022 | Utility of the Total Thrombus‑Formation Analysis System as a Tool  for Evaluating Thrombogenicity and Monitoring Antithrombotic  Therapy in Pediatric Fontan Patients | Japan | Case control | (1)Fontan patients undergoing cardiac catheterization  (2)healthy controls | (1)20  (2)30 | Assessment of thrombogenicity using T-TAS in pediatric fontan patients treated with warfarin and aspirin | PL-chip at 1500 $s^{-1}$  AR-chip at 600 $s^{-1}$ | Conventional coagulation assays | Ability of T-TAS to monitor effect of aspirin and warfarin | $\mathrm{PL}_{18}$-$\mathrm{AUC}_{10}$  $\mathrm{AR}_{10}$-$\mathrm{AUC}_{30}$ | $\mathrm{PL}_{18}$-$\mathrm{AUC}_{10}$  and $\mathrm{AR}_{10}$-$\mathrm{AUC}_{30}$ could be useful to monitor the effects of aspirin and warfarin. |
| Kikuchi et al, 2020 | Platelet-derived thrombogenicity measured by total thrombus-formation analysis system in patients with ST-segment elevation myocardial infarction undergoing primary percutaneous coronary intervention | Japan | Cohort | (1)STEMI patients undergoing PPCI and treated with standard loading dose prasurgel or clopidogrel, aspirin and UFH | (1)127 | Investigation of the time course and value of thrombogenecity measured by T-TAS in STEMI-patients undergoing PPCI compared with VerifyNow | PL-chip at 18 µL/min  AR-chip at 10 µL/min | VerifyNow | Comparison of T-TAS and VerifyNow values in STEMI-patients undergoing PPCI | ${PL}_{18}$-${AUC}_{10}$  ${AR}_{10}$-${AUC}_{30}$ | PRU levels increased during PPCI from baseline, whereas ${PL}_{18}$-${AUC}_{10}$ levels were lower during PPCI compared with baseline.  High ${PL}_{18}$-${AUC}_{10}$ levels during PPCI are associated with large enzymatic infarct size. ${PL}_{18}$-${AUC}_{10}$ can be a suitable marker of platelet derived thrombogenecity during PPCI in STEMI-patients. |

AUC: area under curve, OT: occlusion time, OST: occlusion start time, NR: not reported, PL; platelet, AR: atheroma, T-TAS: total thrombus formation system, ROTEM; Rotational thromboelastometry, DOAC: direct oral anticoagulants, RFCA: Radiofrequency catheter ablation, DAPT: dual antiplatelet therapy, NSTEMI: non–ST-elevation myocardial infarction, LTA: light transmission aggregometry, CT: clotting time, PPCI: primary percutaneous coronary intervention, STEMI: ST-elevation myocardial infarction, PRU: P2Y_12_reaction units.

Table S5: Characteristic features of studies that investigated the role of T-TAS in bleeding risk prediction.

| **First author, year** | **Title** | **Study location** | **Study design** | **Source of study population** | **Sample size** | **Targeted condition** | **Used chip and shear rate** | **Reference standard** | **Analysis used for review** | **Outcome measure(s)** | **Authors conclusion** |
| --- | --- | --- | --- | --- | --- | --- | --- | --- | --- | --- | --- |
| Ito et al, 2016 | Total Thrombus-Formation Analysis System (T-TAS) Can Predict  Periprocedural Bleeding Events in Patients Undergoing Catheter  Ablation (CA) for Atrial Fibrillation | Japan | Cohort | AF patients undergoing CA treated with (1) warfarin and (2) DOACS | (1)30  (2)98 | Monitoring of the anticoagulant effects of DOACs in AF patients undergoing CA | PL-chip at 24 µL/min  AR-chip at 10 µL/min | Conventional coagulation assays | Assessment of thrombogenicity in AF patients treated with DOAC or warfarin and the ability to predict periprocedural bleeding events | $\mathrm{PL}_{24}$-$\mathrm{AUC}_{10}$  $\mathrm{AR}_{10}$-$\mathrm{AUC}_{30}$ | Treatment with anticoagulants significantly decreases $\mathrm{AR}_{10}$-$\mathrm{AUC}_{30}$ and $\mathrm{AR}_{10}$-$\mathrm{AUC}_{30}$ was a significant predictor of periprocedural bleeding events. |
| Nakanishi et al, 2021 | Development and assessment of total thrombus-formation analysis  system-based bleeding risk model in patients undergoing percutaneous  coronary intervention | Japan | Cohort | (1)ARC-HBR positive PCI-patients  (2)ARC-HBR negative PCI-patients | (1)182  (2)118 | Usefullness of T-TAS in prediction of of 1-year bleeding risk in patients undergoing PCI | AR-chip at 600 $s^{-1}$ | NR | Association between T-TAS parameters and 1-year spontaneous bleeding events in PCI-patients | $\mathrm{AR}_{10}$-$\mathrm{AUC}_{30}$ | The combination of ARC-HBR and $\mathrm{AR}_{10}$-$\mathrm{AUC}_{30}$  was useful for predicting 1-year bleeding risk after PCI. |
| Oimatsu et al, 2017 | Total Thrombus‐formation Analysis System Predicts Periprocedural Bleeding Events in Patients With Coronary Artery Disease Undergoing Percutaneous Coronary Intervention | Japan | Cohort | CAD-patients undergoing elevtive percutaneous coronary intervention and treated with DAPT  (1)with periprocedural bleeding  (2)without periprocedural bleeding | (1)276  (2)37 | Evalution of the association of periprocedural bleeding events with thrombogenecity | PL-chip at 2000 $s^{-1}$  AR-chip at 600 $s^{-1}$ | Conventional coagulation assays, Verifynow | Associaton of periprocedural bleeding and thrombogenicity | $\mathrm{AR}_{10}$-$\mathrm{AUC}_{30}$  $\mathrm{PL}_{24}$-$\mathrm{AUC}_{10}$ | $\mathrm{PL}_{24}$-$\mathrm{AUC}_{10}$  levels were significantly lower in patients with periprocedural bleeding events than in those without. PRU and $\mathrm{AR}_{10}$-$\mathrm{AUC}_{30}$.  levels were not significantly different between both groups. |
| Ichikawa et al, 2019 | Impact of Total Antithrombotic Effect on  Bleeding Complications in Patients Receiving  Multiple Antithrombotic Agents | Japan | Cohort | Stable CAD patients treated with oral anticoagulation in addition to single or dual antiplatelet therapy | 145 | Evalution of anti-thrombotic effects and subsequent bleeding complications in patients using mulitple antithrombotic agents | PL-chip  at 18µL/min  AR-chip at 4 µL/min | Conventional coagulation assays | Antithrombotic effect and bleeding risk in patients treated with multiple antithrombotic agents | $\mathrm{PL}_{18}-\mathrm{AUC}_{10}$  $\mathrm{AR}_{4}-\mathrm{AUC}_{30}$ | Patients with bleeding complications had low thrombogenicity compared to patients without bleeding complications. $\mathrm{AR}_{4}-\mathrm{AUC}_{10}$ was an indepent predictor of subsequent bleeding events. |
| Mitsuse et al, 2020 | Total Thrombus-Formation Analysis System can Predict 1-Year Bleeding Events in Patients with Coronary Artery Disease | Japan | Cohort | (1)CAD patients undergoing CAG, treated with DAPT | (1)561 | To predict the 1-year bleeding events in CAD-patients treated with DAPT | PL-chip at 24µL/min  AR-chip at 10µL/min | Conventional coagulation assays | The predictive role of T-TAS values in 1-year bleeding events in CAD-patients | $\mathrm{AR}_{10}$-$\mathrm{AUC}_{30}$  $\mathrm{PL}_{24}$-$\mathrm{AUC}_{10}$ | $\mathrm{AR}_{10}$-$\mathrm{AUC}_{30}$ levels  were lower in bleeding group compared to non-bleeding group, $\mathrm{PL}_{24}$-$\mathrm{AUC}_{10}$  levels were not different between both groups. |

T-TAS: total thrombus formation system, AUC: area under curve, OT: occlusion time, OST: occlusion start time, PL; platelet, AR: atheroma, CAD:

|  |  |  |  |  |  |  |
| --- | --- | --- | --- | --- | --- | --- |
|  |  |  |  |  |  |  |
|  |  |  |  |  |  |  |
|  |  |  |  |  |  |  |
|  |  |  | $m^{2}$  $m^{2}$ |  |  |  |
|  |  |  |  |  |  |  |
|  |  |  |  |  |  |  |
|  | $s^{-1}$  $s^{-1}$  $s^{-1}$ | $s^{-1}$ | $s^{-1}$  $s^{-1}$ | $s^{-1}$ | $s^{-1}$ | $s^{-1}$ |
|  |  |  |  |  |  |  |
|  |  |  |  |  |  |  |
|  |  |  | $\mathrm{PL}_{18}$ $\mathrm{AUC}_{10}$  $\mathrm{AR}_{10}$ $\mathrm{AUC}_{30}$ | $T_{\mathrm{on}}$  $T_{80}$ |  |  |
|  |  |  | $\mathrm{PL}_{18}$ $\mathrm{AUC}_{10}$  $\mathrm{AR}_{10}$ $\mathrm{AUC}_{30}$ | $T_{\mathrm{on}}$ $T_{80}$ |  |  |

[Coronary artery disease, DOAC: direct oral anticoagulant, AF: Atrial Fibrillation, PCI: Percutaneous Coronary Intervention, PRU: P2Y12 reaction units, HBR: high bleeding risk, ARC: Academic Research Consortium, DAPT: Dual antiplatelet therapy, µl/l: microliter per liter, NR: not reported.](https://www.google.com/url?sa=t&rct=j&q=&esrc=s&source=web&cd=&cad=rja&uact=8&ved=2ahUKEwilopynuIuAAxUBgP0HHXJcB2oQFnoECA0QAw&urlTable 6. Characteristic features of studies that investigated role of hemodialysis and thrombocytopenia on T-TAS markers and T-TAS results in COVID-19 patients and endometriosis patients. First author, yearAtari et al, 2020Mitic et al, 2022Nakanishi et al. 2021Ogawa et al, 2013Ghirardello et al, 2021Kedzia et al, 2023TitleA modified microchip-based flow chambersystem for evaluating thrombogenicity inpatients with thrombocytopenia.Platelet thrombus formation in patients with end‑stage renaldisease before and after hemodialysis as measured by the totalthrombus‑formation analysis system.Hemodialysis-related low thrombogenicity measured by totalthrombus-formation analysis system in patients undergoing percutaneouscoronary intervention.Haemodilution-induced changes in coagulation and effectsof haemostatic components under flow conditions.Assessment of Platelet Thrombus Formationunder Flow Conditions in Adult Patients withCOVID-19: An Observational StudyEndometriosis is associated with an increasedwhole-blood thrombogenicity detected by a novelautomated microchip flow-chamber system (T-TAS®)Study locationJapanSerbiaJapanJapanItalyPolandStudy designCohort CohortCohortCase controlCohortCase controlSource of study population (1)patients admitted to the ICU of Kagoshima University hospital requiring a platelet transfusion(1)End stage renal disease (ESDR) patients treated with hemodialysis (HD)Patients undergoing PCI(1)Hemodilution patients(2)non-HD patients eGFR<60 ml/min/1.73(3)non-HD patients eGFR ≥60 ml/min/1.73(1)cardiac patients before/after cardiopulmonary bypass (CPB)(2)healthy volunteers with/without 40% dilution with salineCovid-19 patients admitted to the ICU, subintensive CU and low ICU.Three groups were differentiated(1)early admission(2)intermediate admission(3)late admission(1)Patients with surgically confirmed endometriosis(2)healthy controlsSample size(1)10(1)22(1)33(2)124(3)143(1)15(2)12(1)18(2)19(3)24(1)23(2)10Targeted conditionThe assessment of thrombogenicity in thrombocytopenic patientsEvaluation of T-TAS to study thrombogenicity in ESDR patients before and after HDInvestigation of the relationship between hemodialysis and thrombogenicity in patients undergoing PCIInvestigation of the effects of hemodilution in CPB-patientsEvaluation of T-TAS parameters in different stages of COVID-19Investigate whole blood thrombus formation in patients with endometriosisUsed chip and shear ratePL-chip at 1500 AR-chip at 600 HD-chip at 1100 PL-chip at 1500 PL-chip at 1500 AR-chip at 600 Chip coated with collagen and tissue factor at 1100 and 330 PL-chip at 2000 AR-chip at 240 Reference standardROTEM and Multiplate AnalyzerConventional coagulation assaysNRConventional coagulation assays, ROTEMConventional coagulation assaysConventional coagulation assaysAnalysis used for review(a)Assessment of the relationship between bleeding symptoms and values of T-TAS(b)Assessment of the effect of platelet transfusion on thrombogenicity measured by T-TASPlatelet –dependent thrombogenicity in ESDR patients before and after HDRelation of HD and thrombogenicity in patients undergoing PCIEffect of 40% hemodilution on T-TAS parameters in CPB-patients compared to healthy controlsComparison of platelet-dependent thrombus formation in different stages of COVID-19Comparison of thrombus formation in endometriosis patients vs. healthy controlsOutcome measure(s)AUC HDOT HDPL-AUC--Onset of thrombus formation ()Growth of thrombus formation ()OSTOT AUCOSTOT AUCAuthors conclusion(a)HD chip is able to discriminate between hemostatic function (b)HD chip detects recovery of hemostatic function after platelet transfusion.Platelet thrombogenicity was reduced and below cut-off value for platelet dysfunction in 17/22 patients. HD did not enhance thrombogenicity.Hemodialysis was significantly associated with low -and - levels as measured by T-TAS. and  were prolonged after 40% hemodilution at both flow rates, both in CPB-patients and healthy controls.Platelet thrombus formation was impaired during the first week after admission compared to controls and patients at later disease stages. No difference in platelet thrombus formation was found among patients with different illness severity. Overall, reduced platelet thrombogenicity was observed compared to controls.OST and OT were significantly shorter in patients than in controls. AUC was significantly higher in patients. These results indicate increased thrombogenicity in endometriosis patients.HD: hemodialysis, AUC: area under curve, OT: occlusion time, OST: occlusion start time, PL; platelet, AR: atheroma, T-TAS: total thrombus formation system, s-1: per second, ROTEM; Rotational thromboelastometry, eGFR: estimated glomerular filtration rate, T on: onset of thrombus formation, m2: cubic meter, ICU: intensive care unit, ml: milliliter, CPB: cardiopulmonary bypass, Min: minute, NR: not reported.=https%3A%2F%2Fwww.mayoclinic.org%2Fdiseases-conditions%2Fcoronary-artery-disease%2Fsymptoms-causes%2Fsyc-20350613&usg=AOvVaw3jAiF_K6Mr8epHI9Qm77qT&opi=89978449)

Table S6: Characteristic features of studies that investigated role of hemodialysis and thrombocytopenia on T-TAS markers and thrombogenecity in COVID-19 and endometriosis.

| **First author, year** | **Title** | **Study location** | **Study design** | **Source of study population** | **Sample size** | **Targeted condition** | **Used chip and shear rate** | **Reference standard** | **Analysis used for review** | **Outcome measure(s)** | **Authors conclusion** |
| --- | --- | --- | --- | --- | --- | --- | --- | --- | --- | --- | --- |
| Atari et al, 2020 | A modified microchip-based flow chamber  system for evaluating thrombogenicity in  patients with thrombocytopenia. | Japan | Cohort | (1)patients admitted to the ICU of Kagoshima University hospital requiring a platelet transfusion | (1)10 | The assessment of thrombogenicity in thrombocytopenic patients | PL-chip at 1500 $s^{-1}$  AR-chip at 600 $s^{-1}$  HD-chip at 1100 $s^{-1}$ | ROTEM and Multiplate Analyzer | (a)Assessment of the relationship between bleeding symptoms and values of T-TAS  (b)Assessment of the effect of platelet transfusion on thrombogenicity measured by T-TAS | AUC HD  OT HD | (a)HD chip is able to discriminate between hemostatic function  (b)HD chip detects recovery of hemostatic function after platelet transfusion. |
| Mitic et al, 2022 | Platelet thrombus formation in patients with end‑stage renal  disease before and after hemodialysis as measured by the total  thrombus‑formation analysis system. | Serbia | Cohort | (1)End stage renal disease (ESDR) patients treated with hemodialysis (HD) | (1)22 | Evaluation of T-TAS to study thrombogenicity in ESDR patients before and after HD | PL-chip at 1500 $s^{-1}$ | Conventional coagulation assays | Platelet –dependent thrombogenicity in ESDR patients before and after HD | PL-AUC | Platelet thrombogenicity was reduced and below cut-off value for platelet dysfunction in 17/22 patients. HD did not enhance thrombogenicity. |
| Nakanishi et al. 2021 | Hemodialysis-related low thrombogenicity measured by total  thrombus-formation analysis system in patients undergoing percutaneous  coronary intervention. | Japan | Cohort | Patients undergoing PCI  (1)Hemodilution patients  (2)non-HD patients eGFR<60 ml/min/1.73$m^{2}$  (3)non-HD patients eGFR ≥60 ml/min/1.73$m^{2}$ | (1)33  (2)124  (3)143 | Investigation of the relationship between hemodialysis and thrombogenicity in patients undergoing PCI | PL-chip at 1500 $s^{-1}$  AR-chip at 600 $s^{-1}$ | NR | Relation of HD and thrombogenicity in patients undergoing PCI | $\mathrm{PL}_{18}$-$\mathrm{AUC}_{10}$  $\mathrm{AR}_{10}$-$\mathrm{AUC}_{30}$ | Hemodialysis was significantly associated with low $\mathrm{PL}_{18}$-$\mathrm{AUC}_{10}$  and $\mathrm{AR}_{10}$-$\mathrm{AUC}_{30}$ levels as measured by T-TAS. |
| Ogawa et al, 2013 | Haemodilution-induced changes in coagulation and effects  of haemostatic components under flow conditions. | Japan | Case control | (1)cardiac patients before/after cardiopulmonary bypass (CPB)  (2)healthy volunteers with/without 40% dilution with saline | (1)15  (2)12 | Investigation of the effects of hemodilution in CPB-patients | Chip coated with collagen and tissue factor at 1100 and 330 $s^{-1}$ | Conventional coagulation assays, ROTEM | Effect of 40% hemodilution on T-TAS parameters in CPB-patients compared to healthy controls | Onset of thrombus formation ($T_{\mathrm{on}}$)  Growth of thrombus formation ($T_{80}$) | $T_{\mathrm{on}}$ and $T_{80}$ were prolonged after 40% hemodilution at both flow rates, both in CPB-patients and healthy controls. |
| Ghirardello et al, 2021 | Assessment of Platelet Thrombus Formation  under Flow Conditions in Adult Patients with  COVID-19: An Observational Study | Italy | Cohort | Covid-19 patients admitted to the ICU, subintensive CU and low ICU.  Three groups were differentiated  (1)early admission  (2)intermediate admission  (3)late admission | (1)18  (2)19  (3)24 | Evaluation of T-TAS parameters in different stages of COVID-19 | PL-chip at 2000 $s^{-1}$ | Conventional coagulation assays | Comparison of platelet-dependent thrombus formation in different stages of COVID-19 | OST  OT AUC | Platelet thrombus formation was impaired during the first week after admission compared to controls and patients at later disease stages. No difference in platelet thrombus formation was found among patients with different illness severity. Overall, reduced platelet thrombogenicity was observed compared to controls. |
| Kedzia et al, 2023 | Endometriosis is associated with an increased  whole-blood thrombogenicity detected by a novel  automated microchip flow-chamber system (T-TAS®) | Poland | Case control | (1)Patients with surgically confirmed endometriosis  (2)healthy controls | (1)23  (2)10 | Investigate whole blood thrombus formation in patients with endometriosis | AR-chip at 240 $s^{-1}$ | Conventional coagulation assays | Comparison of thrombus formation in endometriosis patients vs. healthy controls | OST  OT AUC | OST and OT were significantly shorter in patients than in controls. AUC was significantly higher in patients. These results indicate increased thrombogenicity in endometriosis patients. |

HD: hemodialysis, AUC: area under curve, OT: occlusion time, OST: occlusion start time, PL; platelet, AR: atheroma, T-TAS: total thrombus formation system, s^-1^: per second, ROTEM; Rotational thromboelastometry, eGFR: estimated glomerular filtration rate, T _on_: onset of thrombus formation, m^2^: cubic meter, ICU: intensive care unit, ml: milliliter, CPB: cardiopulmonary bypass, Min: minute, NR: not reported, TEG: thromboelastography, COVID-19: coronavirus disease-19, tPA: tissue thromboplastin activator.

Table S7. Reference ranges measured in healthy volunteers in different study groups.

|  |  | **Hosokawa et al. 2011**  **N= 33** | **Daidone et al. 2016**  **N= 20** | **Idemoto et al. 2017**  **N=25** | **Minami et al. 2015**  **N=20** | **Nakajima et al. 2020**  **N= 20** | **Agren et al. 2017**  **N=20** | **Melnichnikova et al. 2022**  **N=33** | **Kedzia et al. 2021**  **N=10** | **Oda et al.**  **2021**  **N=122** |
| --- | --- | --- | --- | --- | --- | --- | --- | --- | --- | --- |
| **AR Chip** |  |  |  |  |  |  |  |  |  |  |
| OST | Min |  | 5, 46 (5,04-6,27) |  |  | 12.1 (10-14.2) | 7,2 (6,8-7,9) |  | 10,65 (4,73-NR) |  |
| OT | Min | 8.8 ± 1.6 | 7,42 (6,54-8,30) |  |  |  | 11,5 (10-12,2) |  | 13,30 (9,99-15,40) |  |
| AUC | /min |  | 1868 (1808-1927) | 1628 (1373-1748) |  | 1221 (1027-1415) | 1657 (1574-1755) |  | 1468.65 (398.35 -NR) |  |
| **PL Chip** |  |  |  |  |  |  |  |  |  |  |
| OST | Min |  | 2,03 (1,04-2,26) |  |  |  |  |  |  |  |
| OT | Min |  | 7,2 (6,11-7,53) |  | 1.45-5.97 | 4.1 (2.8-5.4) |  |  |  |  |
| AUC | /min |  | 878 (784-972) | 342 (278-381) | 123.2-509.2 | 265 (171-359) | 380 (362-419) | 260.5 (217.3–301.9) |  | 385.1 (248-465.6) |

AR-chip: atheroma chip, OST: occlusion starting time, OT: occlusion time, AUC: area under the curve, PL-chip: platelet chip
